# Steganalysis Network for Weak Steganographic Signal Extraction and Enhancement

**DOI:** 10.3390/s26041329

**Published:** 2026-02-19

**Authors:** Weilin Liang, Qingguang Li

**Affiliations:** School of Computer, Electronics and Information, Guangxi University, Nanning 530000, China

**Keywords:** image steganalysis, learnable filters, CNN

## Abstract

The purpose of digital image steganalysis is to identify the signal embedded in the natural image by steganography. In the spatial domain, this embedded signal only modifies the image value of the natural image by ±1, and this modification is weak. However, most of the existing convolutional neural networks use popular components to design or optimize the network structure, without deeply exploring the network’s ability to recognize such weak modifications. In order to deal with this problem, we propose a novel preprocessing structure, the learnable filter constrained by high-pass prior (LFCHP), to improve the network’s ability to extract weak embedded signals in the preprocessing stage, as well as a second-order signal auxiliary branch (SSAB) to reduce the suppression of weak embedded signals during convolution stacking, and a new pooling method, SoftPool, to reduce the loss of weak embedded signals during downsampling. Combining these three structures, we propose a steganalysis network, WSERNet, for weak steganographic signal extraction and enhancement. Experiments conducted under identical conditions demonstrate that the proposed method achieves an accuracy improvement of 1.08–2.96% over state-of-the-art spatial-domain steganalysis algorithms across three steganographic schemes at four embedding rates, and exhibits excellent generalization capabilities across different steganography techniques.

## 1. Introduction

Image steganography is an algorithm that hides information within the redundancy of an image, making it imperceptible to the human eye [1,2,3]. In contrast, image steganalysis is a technique used to detect whether embedded information exists within an image, thus preventing the dissemination of illicit information [4].

Unlike encrypted communication methods [5], existing adaptive image steganography algorithms embed secret information into complex regions of images that are difficult to detect [6,7]. Traditional non-deep learning image steganalysis algorithms usually require hand-designed high-pass filters to extract embedding residuals [8,9,10], but hand-designed filters have limitations [11,12,13,14]. Different from traditional image steganalysis algorithms, image steganalysis algorithms based on deep learning not only reduce dependence on domain knowledge, but also achieve simultaneous optimization of feature extractors and feature classifiers, so more and more image steganalysis algorithms based on deep learning are emerging [15,16,17].

In image steganalysis algorithms, the preprocessing layer is capable of computing noise residuals in complex regions of images, thereby enhancing network performance and accelerating network convergence [18]. Therefore, the preprocessing layer is often employed as a core component in image steganalysis [16,17,19]. However, existing preprocessing layers have their limitations. A typical preprocessing layer design involves using a set of filters from SRM as fixed-parameter convolutional kernels. However, constrained by the limitations of manual design, fixed convolutional kernel parameters are often suboptimal [20]. To mitigate the drawbacks of fixed convolutional kernels, researchers [21,22] have proposed initializing fixed kernels as parameters and optimizing them during network training. However, trainable parameter initialization of filter kernels tends to get trapped in local optima during training, resulting in subpar training outcomes. In order to break free from the dependency and limitations of manual priors, researchers have developed methods [20,23] for completely random initialization. These methods can automatically search for optimal parameters, avoiding the potential harm of manually designed filter kernels to the overall network. However, achieving the ideal scenario of automatically searching for optimal parameters without introducing prior knowledge may result in the network not effectively focusing on the complex areas of image secret message embedding.

Furthermore, in order to ensure the concealment of information in image steganography, the embedded signal only modifies the pixel value of the natural image by ±1, which is very weak. However, both convolution and pooling operations inherently suppress weak signals [24,25], thus potentially weakening the features of these faint steganographic signals during the successive convolutional layers [22,26]. Additionally, in steganalysis, the most commonly used pooling methods are average pooling and global average pooling applied after convolutional layers. However, average pooling assigns equal weights to elements within the kernel neighborhood of feature maps, failing to reinforce the importance of key element values in the feature maps and potentially weakening the features of embedded signals during pooling. Although max pooling may lose significant information and is less commonly used in steganalysis, it retains representative results [27] and should be utilized.

To address the limitations of the aforementioned steganalysis methods, aiming to enhance preprocessing performance and mitigate the adverse effects of convolution and pooling on weak signals, we propose a steganalysis network called WSERNet. Specifically, the contributions of our proposed method are summarized as follows:

- We design a learnable preprocessing structure with high-pass prior constraints. This structure initializes the parameters of the filtering kernel randomly while introducing high-pass prior knowledge through the addition of a high-pass constraint branch. This allows the new network to automatically search for optimal parameters during training while focusing on the complex areas of secret message embedding in images through high-pass prior constraints.
- We introduce a novel auxiliary branch for extracting second-order signals to capture different-order signals, effectively enhancing the features of weak steganographic embedding signals. By utilizing the second-order signal extraction auxiliary branch at multiple levels, we can strengthen the intensity of weak signals, thereby alleviating the suppression of weak signals by convolution. Moreover, these second-order signal auxiliary branches can be removed during inference to avoid complex computations and save time.
- We employ the SoftPool pooling method [27] and extend it to a new global soft pooling (GSP) method. SoftPool pooling can utilize all elements in the kernel neighborhood while assigning greater weights to representative elements, thereby preventing excessive weakening of features of weak embedding signals during pooling. GSP can diversify the compression of features extracted by convolutional layers, better capturing information from feature maps.
- Experimental results demonstrate that WSERNet outperforms the state-of-the-art spatial domain image steganalysis networks in terms of performance and exhibits superior generalization performance across different steganography techniques.

The remaining part of this paper is organized as follows: In Section 2, we review closely related work in spatial domain steganalysis. In Section 3, we provide detailed explanations of the proposed WSERNet. Section 4 validates the effectiveness of the proposed method through extensive experiments. Finally, Section 5 presents the conclusion of this paper and outlines future work.

## 2. Related Works

Tan et al. [15] first applied deep learning methods to image steganalysis. Their network consisted of four convolutional layers, with the first layer parameters multiplied by the KV filter and trained using stacked convolutional autoencoders. Qian et al. [16] proposed the Gaussian-Neuron CNN (GNCNN), which combines a preprocessing layer with convolutional layers. Unlike Tan et al., GNCNN directly employs fixed KV filters as the preprocessing layer and then stacks five convolutional layers. To adapt to modifications in pixel values of stego images, GNCNN introduces a new activation function, the Gaussian activation function, in the convolutional layers to replace ReLU. Xu et al. [17] also introduced Xu-Net, utilizing KV filter kernels and five convolutional layers, but with more detailed designs on the convolutional layers. They applied TanH activation functions in the first and second convolutional layers to constrain activation values and added an absolute layer after the first convolutional layer for subsequent modeling. The last three convolutional layers used convolution with kernel size to limit modeling intensity. Xu-Net achieved performance comparable to the traditional steganalysis method SRM [9]. Ye et al. [18] proposed Ye-Net, an alternative CNN-based method, referring to the three steps of residual calculation, feature extraction, and binary classification in traditional steganalysis methods. Ye-Net widened the convolution in the preprocessing layer from using 1 filter in SRM to 30 filters and introduced a new activation function, the truncated linear unit (TLU), to adapt to the characteristics of embedded signals in the range. Additionally, they designed a method for integrating Selection Channel into the CNN network. Results showed that Ye-Net outperformed traditional methods such as SRM [9] and maxSRM [28] in performance. Boroumand et al. [20] argued that the optimal filter should be learned rather than handcrafted, leading to the proposal of SRNet. SRNet initializes all convolutional kernel weight parameters randomly to avoid manual intervention, disables pooling in shallow convolutions to prevent signal suppression, and adds residual connections to improve detection accuracy. SRNet achieved significant improvements in both spatial and JPEG domains. Zhang et al. [21] introduced a new CNN architecture, Zhu-Net, employing various techniques. Zhu-Net standardized the sizes of 30 convolutional kernels in the preprocessing layer and initialized them using the basic filters from SRM [9]. During training, the preprocessing kernels were also optimized. Additionally, Zhu-Net utilized two depthwise separable convolution blocks in the shallow layers and applied spatial pyramid pooling [29] after the convolutional layers to compress feature maps.Deng et al. [19] proposed a Covariance Pooling Steganalysis Network (CPSN). CPSN designed a fast and effective network architecture through extensive experiments and further improved network performance using covariance pooling [30], achieving better detection results than SRNet. You et al. [31] introduced SiaStegNet, capable of detecting images of arbitrary sizes. SiaStegNet divided the input image into two subregions, each inputted into one of the two subnetworks of Siamese [32]. The outputs of each subnetwork were concatenated to distinguish between cover and stego results. Moreover, the outputs of each subnetwork were compared using contrastive loss [33] to extract the similarity between the two subregions, adapting better to images of arbitrary sizes. Liu et al. [22] explored methods to reduce the suppression of weak signals embedded during convolution and proposed FPNet. FPNet introduced the efficient significant expansion front part (FEPM) to enhance the transmission of weak signals and designed the attention downsampling module (ADM) to mitigate the suppression of weak signals by pooling. Weng et al. [34] presented the lightweight LWENet, which utilized 30 fixed filter kernels from SRM, followed by bottleneck residual blocks (BRBs) to further improve the residual signal-to-noise ratio. LWENet also employed multi-view global pooling (MGP) and depthwise separable convolution (DWSConv) techniques to reduce parameter count while enhancing detection performance.

## 3. Materials and Methods

To enhance the accuracy of image steganalysis by effectively identifying weak embedded signals, this paper proposes a convolutional neural network named “WSERNet.”

### 3.1. Overview of Method

**Figure 1:**
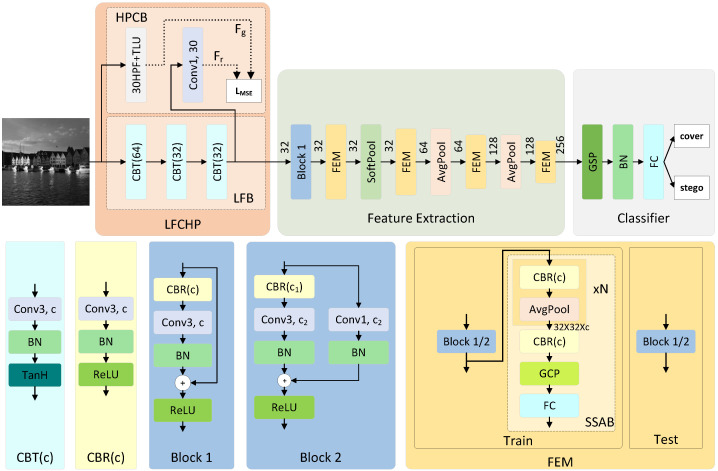
The overall structure of WSERNet. WSERNet consists of three parts: LFCHP, Feature Extraction, and Classifier. LFCHP includes LFB and HPCB. LFB eliminates the defects of fixed convolution kernels through random initialization. In HPCB, 30HPF+TLU is a commonly used preprocessing method, its output is $F_{g}$, and the number of channels is 30. “Conv1, 30” changes the number of output channels of LFB from 32 to 30, aligning the number of channels of $F_{r}$ and $F_{g}$. Through $L_{MSE}$, HPCB is responsible for imposing high-pass prior constraints on LFB to keep LFB focused on complex areas. SSAB, a second-order signal auxiliary branch in FEM, enhances weak signal features using second-order statistical information from feature maps. The numbers in Feature Extraction and CBT (c) represent the number of channels. “Block 1/2” indicates the option to choose between Block 1 and Block 2, with Block 1 used when the input and output channel numbers are equal, and Block 2 used otherwise. “(Conv1/3, c)” denotes convolutional layers with a kernel size of 1 or 3 and output channel numbers of c. During the inference phase, all HPCB and SSAB branches are removed.

As shown in Figure 1, the proposed WSERNet consists of three parts: (1) the preprocessing part, represented by the LFCHP (learnable filters constrained by high-pass prior) module in Figure 1, composed of a high-pass prior constraint branch (HPCB) and a learnable filter bank (LFB), responsible for preprocessing the input image; (2) the Feature Extraction part, comprising one Block 1 module, four FEMs (Feature Enhancement Modules), one SoftPool, and two AvgPool; (3) the classifier, composed of GSP (Global SoftPool), BN (Batch Normalization); and a fully connected layer (FC). The network structure of WSERNet differs between the training and inference stages. In the training stage, LFCHP includes HPCB, and each FEM contains a second-order signal auxiliary branch (SSAB). In the inference stage, neither HPCB nor SSAB affects the results of the main network; thus, these branch structures are removed.

### 3.2. The LFCHP Module: Learnable Filters with High-Pass Prior Constraint

In the preprocessing stage, existing methods usually use manually designed filter kernels or randomly initialize the convolution kernel. Manually designed filter kernels can strictly focus on complex areas where information is embedded, but manual experience cannot represent all situations, and the feature extraction is not completely enough. The method of randomly initializing the convolution kernel automatically searches for the optimal parameters in theory, but the CNN pays more attention to mid-frequency and low-frequency information, and does not pay enough attention to high-frequency information [35], that is, the complex area where information is embedded. In order to combine the advantages of the two methods, we propose learnable filters constrained by high-pass prior, as shown in the LFCHP part in Figure 1, which consists of two parts: LFB and HPCB.

The LFB part consists of three CBT (c) modules, where c denotes the number of output channels. The parameters of the convolutional layers in each CBT module are randomly initialized, allowing each convolutional kernel to automatically search for the optimal parameters. The output channel numbers of the three CBT modules are 64, 32, and 32. The first convolutional module with 64 channels is used to extract more diversified features directly from the input image. For spatial image steganography, embedding secret information is achieved by modifying the grayscale values of the carrier image pixels. In this case, using the ReLU activation function may lead to the loss of a considerable amount of information. Therefore, we choose the hyperbolic tangent function (TanH) as the activation function to better adapt to the characteristics of the steganographic embedding signal.

In the HPCB part, adaptive steganography embeds secret information into the complex high-frequency areas of images. Therefore, we need to encourage the steganalysis network to focus on high-frequency information. The loss function enables the network to learn to target, while the high-pass filter can strictly focus on the high-frequency area, but the high-pass filter is difficult to use as a direct learning target. Therefore, we use the output image of the high-pass filter as the learning target; that is, $F_{r}$ learns from $F_{g}$. The high-pass filter uses the classic setting [19]. Specifically, we use 30 filters from SRM as an additional branch, with each filter normalized and the filter weights fixed. Then, a linear truncation activation function (TLU) with a threshold of 3 is used to filter out large-value elements with low signal-to-noise ratios. After TLU, an output $F_{g}$ is formed with a channel number of 30, serving as one input to Equation (1). Additionally, the number of output channels of LFB is 32, which is inconsistent with the target number of 30 channels. Therefore, the number of channels needs to be adjusted; that is, a convolution layer branch with a convolution kernel size of 1 and an output channel number of 30 is introduced after the LFB to form the output $F_{r}$, which is another input to Equation (1). This convolutional layer does not have batch normalization (BN) and activation functions. In addition to adjusting the number of channels, adding this convolutional layer branch also allows each channel of the third CBT module to learn from all high-pass filters, so that the convolution kernel in the LFB has richer prior knowledge. Finally, $F_{r}$ and $F_{g}$ are entered into the mean square error loss function $L_{MSE}$, and $F_{g}$ is constrained to $F_{r}$ by $L_{MSE}$; that is, $F_{r}$ learns from $F_{g}$, where the eigengraph size of $F_{r}$ and $F_{g}$ is M·N, and the loss function $L_{MSE}$ is defined as

(1) $$L_{MSE}=\frac{1}{M\cdot N}\sum_{i=1}^{M}\sum_{i=1}^{N}{\left(F_{r}\left(i,j\right)-F_{g}\left(i,j\right)\right)}^{2}$$

### 3.3. Feature Enhancement Module

Since the embedding signal is distributed in the high-frequency area of the image, this will cause the weak embedding signal to disappear during the convolution stacking process. Therefore, we need to maintain attention to the high-frequency area in the network. The greater the difference in adjacent element values of the feature map, the more dispersed the distribution of element values will naturally be, and the calculated element value variance will usually be higher. Then, using variance and covariance in the network will make the network pay more attention to high-frequency areas. GCP can calculate the variance of the feature map itself and the covariance between different feature maps, that is, the second-order information of the feature map. Therefore, using GCP can enable the network to maintain focus on high-frequency areas, thus avoiding the problem of weak embedded signals disappearing during the convolution stacking process. We drew on the usage of GCP in the image classification method Detachable Second-Order Pooling (DSOP) [36], proposed a method called SSAB, and built a weak signal feature enhancement module (FEM).

Each FEM comprises a Block 1/2 and an SSAB branch. Block 1/2 indicates the option to choose between Block 1 and Block 2 (Block 1 is used when the input and output channel numbers are equal, and Block 2 is used otherwise).

In image steganalysis tasks, downsampling is typically disabled in shallow convolutions to avoid signal suppression. Moreover, due to computational constraints, the channel numbers of shallow feature maps cannot be excessive. These factors often result in shallow feature maps characterized by high resolution and low channel numbers. Based on this feature, we improved the structure of DSOP and named it SSAB, as illustrated by the SSAB of FEM in Figure 1.

SSAB initially reduces the feature map size of the input with a size of H×W×c using N CBR (Conv+BN+ReLU) and AvgPool layers until the output feature map size becomes 32×32, i.e., $\left(H/2^{N}\right)\times \left(W/2^{N}\right)=32\times 32$. Therefore, the sizes of N in the 4 FEMs are 3, 2, 1, and 0 in order. Subsequently, the integrated average-pooled information is processed by a CBR. The channel numbers remain unchanged throughout these processes. After dimension reduction of the feature map size, global covariance pooling (GCP) is employed to extract global second-order information, preserving only the channel dimension. Finally, classification and training are performed through a fully connected layer. Specifically, each SSAB branch is equipped with a classifier for convenient training of SSAB.

Block 1 consists of a CBR, Convolution, BN, residual connection, and ReLU. In contrast to Block 1, the residual connection part of Block 2 contains Convolution and BN for adjusting the channel number.

We apply FEM hierarchically in the network, reinforcing weak signal features in multiple stages to effectively prevent signal loss. Moreover, all SSAB branches are removed during the inference phase, avoiding an increase in computational costs during actual network applications.

The loss function used for each FEM during training is cross-entropy loss ($L_{CE,n},n=1,2,3,4$). The total loss function $L_{FEM}$ across the four FEMs is defined by the following Equation (2):

(2) $$L_{FEM}=\sum_{i=1}^{4}L_{CE,n}$$

Each $L_{CE,n}$ corresponds to a different loss function in the different FEMs.

The loss function used for the classifiers is cross-entropy loss $L_{CE,clf}$. Since both the FEMs and classifiers use cross-entropy loss, we do not introduce additional weighting hyperparameters for $L_{FEM}$. Therefore, the total loss function is given by the following Equation (3):

(3) $$L=L_{CE,clf}+\lambda \cdot L_{MSE}+L_{FEM}$$

### 3.4. SoftPool and Global SoftPool (GSP)

For most CNN-based steganalysis networks, average pooling is typically employed between convolutional layers. Pooling reduces the model size, improves computational speed, and enhances robustness. However, conventional pooling methods may not adapt well to the characteristics of steganalysis, leading to weakening of the embedded signals.

To address the limitations of conventional pooling layers, we utilize SoftPool [27] for feature map downsampling in the network. The calculation method is illustrated by the following Equation (4):

(4) $$\tilde{a}=\sum_{i\in R_{0}}\frac{e^{a_{i}}}{\sum_{j\in R}e^{a_{j}}}a_{i}$$

In this context, $a_{i}$ represents an element in the feature map, $R_{0}$ denotes the kernel neighborhood for downsampling, and $\tilde{a}$ signifies the sampling result. SoftPool offers several advantages: compared with average pooling, it can effectively highlight important features; compared with max pooling, it avoids discarding excessive information, making it better suited for steganalysis by preserving critical features more effectively during the pooling process.

Additionally, we employ SoftPool as the Global SoftPool (GSP) after the final convolution module, enhancing its diversity by introducing a coefficient. Its expression is depicted in the following Equation (5):

(5) $$\tilde{a}=\sum_{i\in R}\frac{e^{na_{i}}}{\sum_{j\in R}e^{na_{j}}}a_{i}$$

At this point, *R* represents the entire feature map, and *n* denotes the coefficient being added. We set *n* to four different values: n=−1,0,1,+∞. When n=−1, it emphasizes weaker elements; when n=0, it is equivalent to global average pooling (GAP), emphasizing all elements equally; when n=1, it highlights representative elements while also integrating others; and when n=+∞, it is equivalent to global max pooling (GMP), only emphasizing representative elements. This configuration of GSP enables a comprehensive assessment of the feature map’s characteristics, facilitating a better collection of information from the feature map.

## 4. Experimental Results and Analysis

### 4.1. Dataset and Environment Settings

In this section, we conducted extensive experiments to demonstrate the effectiveness of WERSNet. The datasets used in our experiments are BOSSbase 1.01 [37] and BOWS2 [38], each containing 10,000 grayscale images of size 512×512. Due to limitations in GPU computational capacity, we resized the image resolution from 512×512 to 256×256. We use steganography to generate stego images after the image resizing is completed. Therefore, the operation of resizing the image will not have a potential impact on the experimental results.

We employed three different spatial domain image steganography methods in our experiments: WOW [11], S-UNIWARD [39], and HILL [12]. Each steganography method includes four different payload sizes: 0.1 bpp, 0.2 bpp, 0.3 bpp, and 0.4 bpp. For each steganography method, each embedding rate generated 10,000 cover-stego pairs. The dataset used in Section 4.2, Section 4.3 and Section 4.4 is BOSSbase, with a training–validation–testing split ratio of 4:1:5. Each sample pair is randomly partitioned. Additionally, in Section 4.2, Section 4.3 and Section 4.4, only BOSSbase 1.01 is used. In Section 4.5, the training set also added 10,000 pairs of images from BOSW2.

All experiments employed two data augmentations: random horizontal flipping and random rotation at four angles ($0^{\circ },90^{\circ },180^{\circ },270^{\circ }$). The optimizer used during training is SGD, with weight decay and momentum values set to 0.0005 and 0.9, respectively. The batch size is 32 (16 pairs of cover-stego). L2 regularization is disabled for all convolutional and fully connected layers’ bias terms. The initial learning rate is 0.01, divided by 10 at epochs 80, 140, and 180, for a total of 300 epochs. To ensure fair comparison, all steganalysis models were implemented using PyTorch on identical hardware facilities. All experiments were repeated three times, and the average value was taken as the final result. The GPU used is NVIDIA RTX3090, with Python version 3.7 and PyTorch version 1.7.1.

### 4.2. Ablation Study of LFCHP

LFCHP is a newly designed preprocessing structure for steganalysis that we have developed. In this section, we will examine the effectiveness of LFCHP. Initially, we will consider the impact of hyperparameters λ in Equation (3) on the entire network. The results in Table 1 show that when the hyperparameter λ=0.1, the detection accuracy is 85.25%, achieving the best detection performance. We will set λ=0.1 in all subsequent experiments. For other cases, detection performance is weaker but still yields good results. We attempted to set λ=0, meaning not introducing high-pass prior knowledge, as a comparative experiment with other values. However, in this scenario, WERSNet fails to converge.

**Figure 2:**
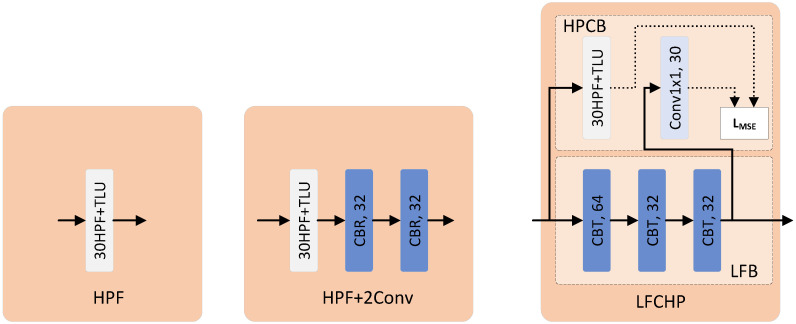
Different preprocessing layers. HPF is the classical preprocessing method. HPF+2Conv adds two convolution layers after HPF for comparison with LFCHP. LFCHP is our proposed preprocessing method.

To further investigate the enhancement of detection performance by LFCHP, we compared several different preprocessing approaches, as shown in Figure 2.

HPF in Figure 2 is composed of a convolution layer and TLU (truncation threshold T=3) activation function, where the parameters of the convolution layer are initialized and fixed by 30 different high-pass filters in SRM, which can effectively filter out low-frequency signals and enhance the signal-to-noise ratio of embedded signals, and is a classic preprocessing layer structure in steganography.

LFCHP in Figure 2 is our proposed preprocessing structure. LFB is randomly initialized and trained jointly by dataset and HPCB, which not only solves the possible defects of manual design, but also integrates high-pass prior knowledge and enables the preprocessing layer to focus on the complex region where the embedded signal is located.

The preprocessing layer scheme in Figure 2b is used as an ablation scheme. As can be seen from Table 2, HPF+2conv only improves by 0.09% and 0.18% compared to HPF, indicating that blindly adding convolutional layers cannot significantly improve the performance of the network, while our proposed LFCHP improves by 1.97% and 2.00% compared with HPF, which is a significant improvement, indicating that the improvement achieved by LFCHP is not caused by the additional two layers of convolutional layers, reflecting the rationality and superiority of the LFCHP design.

**Figure 3:**
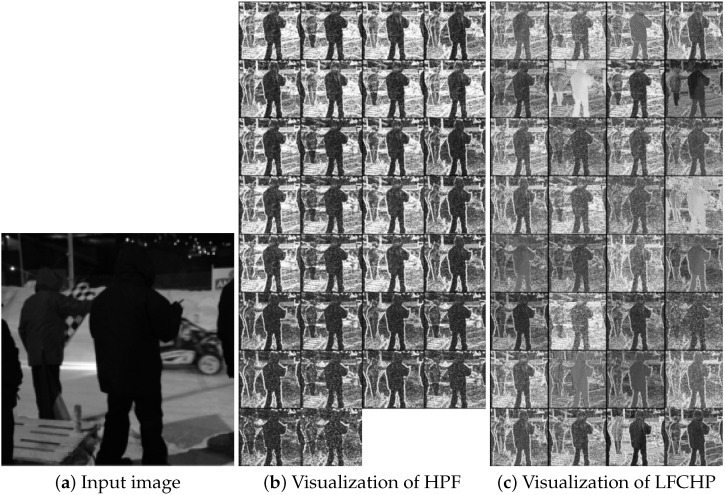
Visualization of different preprocessing results. (**a**) is the input image. (**b**) is the result image obtained after the input image has been preprocessed by 30HPF+TLU. The image difference is not significant and has high redundancy. (**c**) shows the result image obtained after LFCHP preprocessing of the input image. The image difference is more obvious, reducing information redundancy, and the focus on complex areas is also maintained.

To more intuitively demonstrate the advantages of our proposed LFCHP, we visualized the output results of two preprocessing layers, HPF (a high-pass filter with 30 fixed parameters) and LFCHP (32 channels in the output feature map) in Figure 2, as shown in Figure 3. Compared with HPF, LFCHP offers the following advantages:

(1) After HPF processing, many points are truncated by the TLU activation function (depicted as white points in Figure 3c). These truncated pixels have relatively low signal-to-noise ratios. While truncating these points helps the network focus on high signal-to-noise ratio points and aids in network training, it also results in loss of embedded information, potentially impacting network performance improvement. LFCHP, after optimization through training, adapts better to the characteristics of the embedding regions. Consequently, fewer points are forcibly truncated after LFCHP processing, indicating that LFCHP preserves more embedded information compared with HPF.

(2) The grayscale values of the filtering results after HPF processing are limited to a few fixed amplitudes, potentially causing the network to overlook many details. In contrast, the grayscale values of the feature maps after LFCHP are not limited to a few values; they are more evenly distributed and comprehensive, enabling more detailed extraction of embedded signal features.

(3) The variations between different filtering results after HPF processing are not very distinct, resulting in considerable information redundancy. In contrast, LFCHP exhibits significant variations between different channels, leading to richer feature extraction and significantly reduced information redundancy.

### 4.3. Ablation Study for the SSAB and the Pooling Layer

For the issue of weak signal features possibly disappearing as they propagate from shallow to deep networks, existing methods typically employ residual connections to aid feature propagation. In addition to residual connections, we also considered methods to enhance the strength of weak signal features. To validate this idea, we removed SSAB from WERSNet while keeping the remaining structure unchanged, and then compared it with the cover WERSNet. The results in Table 3 indicate that after removing SSAB, WERSNet’s performance decreased by 1.21% and 1.12% at payload rates of 0.2 bpp and 0.4 bpp, respectively.

The faintness of steganographic signals is an inherent property for security reasons, yet it also leads to the suppression of weak signals during convolutional stacking in steganalysis. Therefore, enhancing the features of weak signals can effectively improve classification performance. Through SSAB, we extract second-order information such as variance and covariance from the network, which can better capture subtle variations in feature maps, thus fully extracting the features of weak signals. By constructing FEM with SSAB and hierarchically applying FEM to WERSNet, we can enhance the features of weak signals at multiple levels, mitigating the inhibitory effect on weak signals during convolutional stacking.

Due to the fact that common pooling methods tend to suppress the features of weak signals, we attempted different combinations of pooling and global pooling to alleviate this issue, as shown in Table 4. Experimental results show that the accuracy of MaxPool+GAP is 82.78%, which is the lowest among all methods. This is because MaxPool will lose a lot of information, resulting in a significant decrease in performance. The accuracy of AvgPool+GAP is 84.31%, which is significantly improved compared with MaxPool+GAP. This is because AvgPool can utilize all elements in the kernel field, which alleviates the problem of excessive information loss to a certain extent, but it is still falls short. SoftPool, building upon the utilization of kernel field information, emphasizes key elements, further enhancing performance; specifically, the combination of SoftPool+GAP outperforms AvgPool+GAP by 0.39%. Though the performance gain is not substantial, it does not entail additional parameter overhead.

In the Feature Extraction section, replacing the other two AvgPools with SoftPool did not yield further performance improvements; hence, we refrain from replacing these AvgPools. Furthermore, the extension of SoftPool to GSP achieved a notable performance boost. In summary, the combination of SoftPool+GSP improves performance by 0.94% compared with AvgPool+GAP and by 2.47% compared with MaxPool+GAP. This suggests that the new pooling methods effectively alleviate the inhibitory effect on weak signal features during network propagation compared with existing pooling methods, thereby enhancing network performance. SoftPool not only utilizes all information within the kernel neighborhood but also emphasizes representative features, reducing information loss during downsampling. GSP, building upon SoftPool, captures feature map information from multiple perspectives, enriching the features of global pooling.

### 4.4. Comparison of Generalization Performance Across Different Steganography Techniques

To assess the generalization performance of WERSNet, we conducted experiments with mismatched steganographic sources. In this section of experiments, the steganalysis methods used were trained on a single steganographic method’s training set and tested on test sets with different steganographic methods, all constructed with a payload of 0.4 bpp.

Table 5 presents the results of WERSNet compared with SRNet, Zhu-Net, CPSN, FPNet, and LWENet in detecting mismatched steganographic sources under three steganographic techniques. Overall, all detection results of WERSNet outperform those of other steganalysis networks. When the test set employs the easiest-to-detect method, WOW, all steganalysis networks demonstrate good generalization performance. For a training set using HILL and a test set using WOW, all steganalysis networks show higher detection accuracy compared with when the test set uses HILL; however, our model’s detection accuracy is significantly superior to other networks. With the test set using S-UNIWARD, all steganalysis networks experience varying degrees of performance loss, with ours exhibiting the lowest loss. When the test set utilizes the most challenging to detect, HILL, our model’s performance loss is lower than FPNet and LWENet, higher than SRNet and Zhu-Net, comparable to CPSN, yet still superior to other methods in terms of detection accuracy. Overall, our model outperforms the other five methods in terms of generalization performance across different steganography techniques, making it more suitable for practical applications.

### 4.5. Performance Comparison

To validate the effectiveness of WERSNet, we conducted a fair comparison with five state-of-the-art steganalysis networks, including SRNet, Zhu-Net, CPSN, FPNet, and LWENet. In addition to the BOSSbase dataset, we added the BOWS2 dataset to ensure better representation of different methods’ performance. The steganographic algorithms employed were WOW, S-UNIWARD, and HILL, with each steganalysis algorithm using the same dataset split (as detailed in Section 4.1). Each steganographic algorithm utilized payloads ranging from 0.1 bpp to 0.4 bpp.

**Figure 4:**
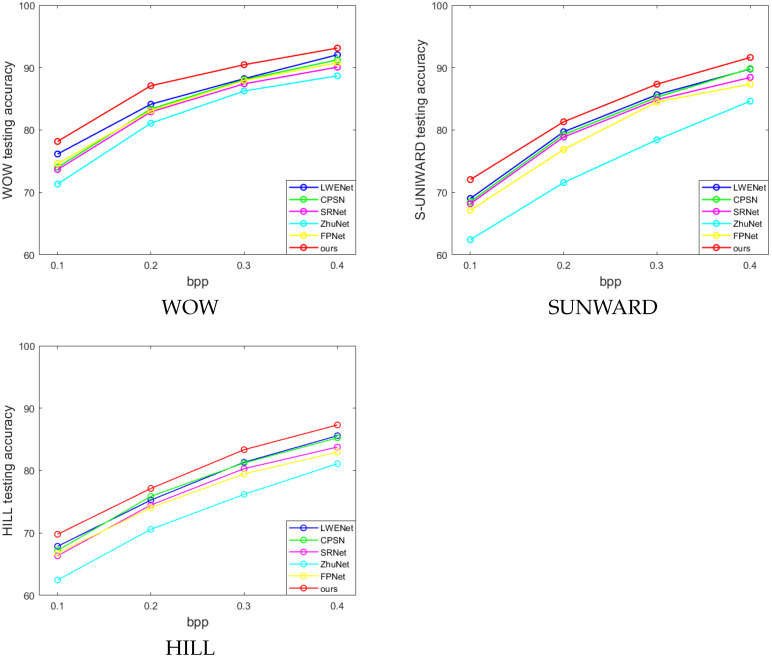
WERSNet comparison of performances with SRNet, Zhu-Net, CPSN, FPNet, and LWENet under the three steganographic algorithms.

The experimental results presented in Table 6 and Figure 4 demonstrate that for any steganographic algorithm and payload used, WERSNet achieved higher detection accuracy compared with the other five steganalysis networks. Specifically, WERSNet exhibited a 2.45% to 4.51% improvement over SRNet, a 6.22% to 9.55% improvement over Zhu-Net, a 1.26% to 4.14% improvement over CPSN, a 2.38% to 4.92% improvement over FPNet, and a 1.08% to 2.96% improvement over LWENet.

**Figure 5:**
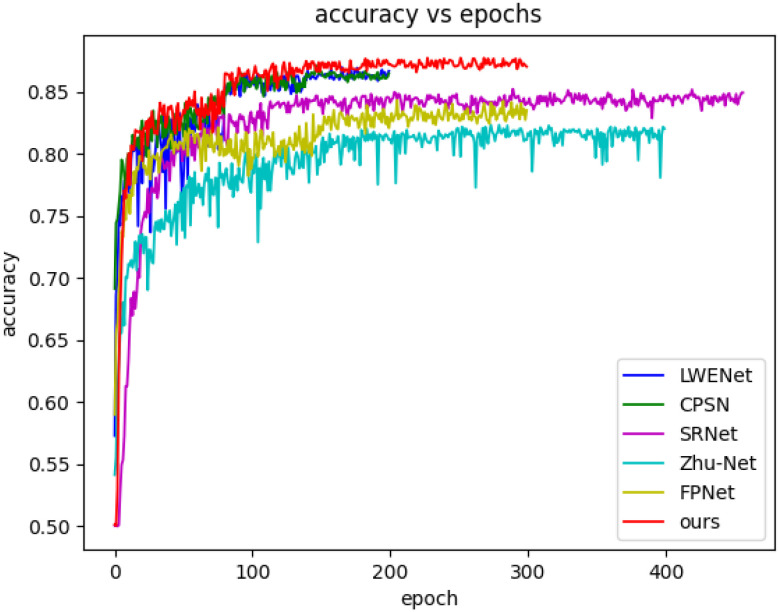
The accuracy curves of six networks on the validation set under HILL at 0.4 bpp.

Additionally, we depicted the validation set accuracy curves for these six methods with a payload of 0.4 bpp, as shown in Figure 5. The validation set accuracy curves accurately reflect the convergence status of various methods. WERSNet exhibited convergence speeds similar to CPSN and LWENet, both of which utilize fixed high-pass filtering kernels, but with superior accuracy performance. These three methods converged faster than FPNet, Zhu-Net, and SRNet, which employ trainable high-pass filtering kernels and randomly initialized kernels. This observation highlights that LFCHP not only demonstrates excellent performance in accuracy but also exhibits competitive convergence speeds.

## 5. Conclusions

In this paper, we introduce a convolutional neural network called WERSNet for steganalysis of spatial domain images. Starting from enhancing weak steganographic signal features, we design a novel preprocessing structure, LFCHP, which significantly improves preprocessing performance by introducing high-pass prior constraints while being randomly initialized. Furthermore, utilizing SSAB, we construct an FEM capable of enhancing weak signal features and hierarchically apply FEM to WERSNet, thereby enhancing weak signal features at multiple levels and alleviating the suppression effect on weak signals during convolution stacking. Additionally, we replace some AvgPool operations with SoftPool and extend SoftPool to GSP. SoftPool emphasizes representative features while utilizing information from the entire kernel neighborhood, reducing information loss during downsampling. GSP, based on SoftPool, captures feature map information from multiple angles, enriching global pooling features. Compared with existing steganalysis networks, WERSNet achieves significant improvements and demonstrates outstanding generalization performance across different steganography techniques.

In future work, we will explore the application of LFCHP in various steganalysis domains such as text and audio steganalysis. Additionally, we will investigate methods to enhance the generalization performance of steganalysis networks, which is crucial for practical application scenarios.

## Figures and Tables

**Table 1:** Detection accuracy (%) of WERSNet under different *λ* values.

*λ*	0.07	0.08	0.09	0.1	0.2	0.3	0.4
Acc	85.07	84.87	85.20	85.25	85.10	85.14	85.02

**Table 2:** Detection accuracy of different preprocessing layer schemes.

Preprocessing	HILL (0.2 bpp)	HILL (0.4 bpp)
HPF	73.40	83.25
HPF+2conv	73.49	83.43
LFCHP	75.37	85.25

**Table 3:** Ablation experiment of SSAB.

Network	HILL (0.2 bpp)	HILL (0.4 bpp)
WSERNet without SSAB	74.16	84.13
WSERNet with SSAB	75.37	85.25

**Table 4:** Impact of different pooling methods on performance with the HILL embedding method at a payload of 0.4 bpp.

Pooling+Global Pooling	Accuracy	Number of Classifier Parameters
MaxPool+GAP	82.78	514
AvgPool+GAP	84.31	514
SoftPool+GAP	84.70	514
AvgPool+GSP	84.89	2050
SoftPool+GSP	85.25	2050

**Table 5:** The experiments of mismatched steganographic sources on the BOSSbase dataset.

Network	Train Method	Test Method		
		WOW	S-UNIWARD	HILL
SRNet	WOW	87.41	78.39	65.88
	S-UNIWARD	84.12	85.19	73.41
	HILL	81.83	73.43	81.59
Zhu-Net	WOW	85.74	76.27	63.82
	S-UNIWARD	81.76	82.53	67.42
	HILL	80.11	72.84	79.26
CPSN	WOW	89.51	82.44	65.42
	S-UNIWARD	88.84	88.39	73.18
	HILL	84.10	76.72	83.34
FPNet	WOW	88.12	75.30	62.51
	S-UNIWARD	83.48	86.17	67.79
	HILL	81.18	73.60	80.09
LWENet	WOW	89.83	81.10	64.62
	S-UNIWARD	88.73	88.56	71.98
	HILL	83.97	77.11	83.58
ours	WOW	91.43	84.72	67.47
	S-UNIWARD	90.41	89.90	73.67
	HILL	86.02	80.41	85.25

**Table 6:** Comparison of accuracy (Acc) of six methods under three steganographic techniques at four embedding rates.

Network	WOW				S-UNIWARD				HILL			
	0.1	0.2	0.3	0.4	0.1	0.2	0.3	0.4	0.1	0.2	0.3	0.4
SRNet [20]	73.67	82.91	87.41	90.08	68.20	78.88	84.86	88.43	66.34	74.49	80.31	83.78
Zhu-Net [21]	71.34	81.10	86.25	88.69	62.43	71.58	78.44	84.63	62.46	70.59	76.20	81.10
CPSN [19]	74.04	83.37	88.07	91.25	68.52	79.26	85.25	89.85	67.20	75.88	81.22	85.25
FPNet [22]	74.63	83.09	87.95	90.76	67.14	76.88	84.50	87.36	66.75	74.07	79.47	82.96
LWENet [34]	76.16	84.14	88.23	92.06	69.03	79.72	85.66	89.78	67.87	75.27	81.34	85.59
Ours	78.18	87.10	90.47	93.14	72.06	81.33	87.35	91.63	69.79	77.14	83.35	87.32

## Data Availability

Code available: https://github.com/LWL139/WSERNet green(accessed on: 5 February 2026). Additional data are available upon request to the corresponding author.

## Abbreviations

The following abbreviations are used in this manuscript:

LFCHP	learnable filter constrained by high-pass prior
SSAB	second-order signal auxiliary branch
CNN	convolutional neural network
GNCNN	Gaussian-Neuron CNN
TLU	truncated linear unit
CPSN	Covariance Pooling Steganalysis Network
HPCB	high-pass prior constraint branch
LFB	learnable filter bank
FEM	feature enhancement module
BN	batch normalization
FC	fully connected
TanH	hyperbolic tangent function
GSP	global Soft Pooling
GCP	global covariance pooling
GAP	global average pooling
GMP	global max pooling
